# “Turn on” fluorescence sensors sensitive to volatile organic solvents/plasticizers -perspective and challenges

**DOI:** 10.3389/fchem.2025.1710573

**Published:** 2025-11-18

**Authors:** Justyna Kalisz, Krzysztof Maksymiuk, Agata Michalska, Emilia Stelmach

**Affiliations:** Faculty of Chemistry, University of Warsaw, Warsaw, Poland

**Keywords:** fluorimetric sensors, plasticizers, volatile organic compounds, polymeric nanoparticles, electrospun nanofibers

## Abstract

Organic liquids immiscible with water, such as volatile organic compounds (VOCs) and plasticizers are widespread harmful environmental pollutants, which have long been considered a risk factor for chronic diseases in humans. VOCs or plasticizers in the outdoor environment largely originate from industrial manufacturing, combustion and leakage of transportation fuels, and biological metabolism. Consequently, in order to protect the environment and human health, there is an urgent need for point-of-need sensors for VOCs as well as plasticizer detection. Fluorimetric sensors are emerging attractive tool for monitoring concentration changes of these analytes in environmental samples, that can reduce need for advanced, instrumental methodologies. Various fluorescence-based strategies have already been demonstrated, with turn-on fluorescence approaches being particularly promising. These strategies are based on the interaction of fluorometric dyes or conjugated polymers embedded in polymeric matrices with the target analytes. The proposed approaches show great potential for real-time monitoring of hazardous pollutants in environmental applications, offering cost-efficient, simple, and portable alternatives to conventional analytical techniques. However, it is worth emphasizing that despite the great variety of research topics, the current state of knowledge does not exhaust this field, and many challenges still remain to be overcome.

## 1 Introduction

**FIGURE 1:**
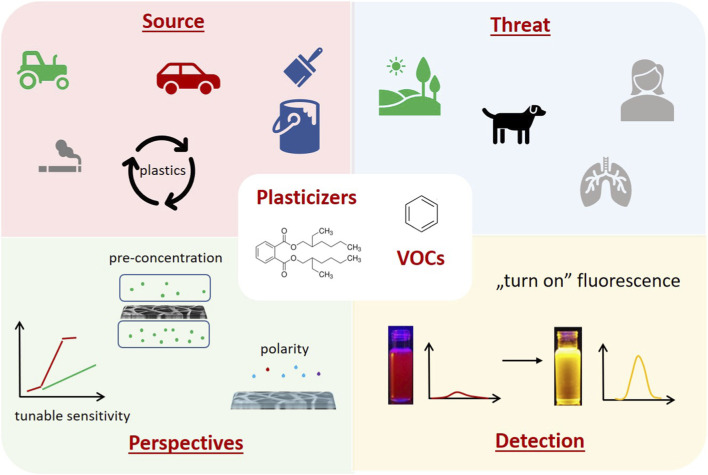
Schematic representation of the idea of this work.

Environmental contamination, especially water pollution, is a significant global concern, and the shortage of fresh water is one of the greatest challenges of modern civilization (Grzegorzek et al., 2023). Nowadays, the environment is increasingly polluted by industrial effluents, agricultural leaching, and the growing amounts of domestic pollutants resulting from population growth (Ejiohuo et al., 2025) (Figure 1). Considering the growing public awareness of the threats posed by the presence of toxic substances in the environment, it is particularly important to search for alternative methods of their detection. Among these pollutants, some of the most hazardous are (VOCs) (Baskaran et al., 2024) and plasticizers (Flora et al., 2025) (Figure 1). VOCs and plasticizers - especially those immiscible with water - exhibit low chemical reactivity, which makes their selective detection challenging.

The golden standard method for VOC detection is gas chromatography (GC), often preceded by a microextraction step. This technique has long been considered the reference method for analyzing the presence and distribution of VOCs in environmental samples (Chary and Fernandez-Alba, 2012; Marć et al., 2012; Solórzano-García et al., 2025). Porous materials such as metal–organic frameworks (MOFs) or semiconductor materials have also been proven effective as sensors for detecting VOCs in the gas phase, using measurements of electrical resistance or other electrical parameters as the detection mode (Chen et al., 2025; Khatib and Haick, 2022). Compared to traditional instrumental analytical methods, chemical gas sensors are relatively inexpensive and allow real - time monitoring (Wang et al., 2018). However, the main drawback of these devices is their high operating temperature - often exceeding 200 °C - which limits their applicability under field conditions. For economic and thus practical reasons, electronic nose (e-nose) technology appears to be a promising approach for VOC detection (Li et al., 2024). Such devices consist of an array of multiple low-cost sensors, providing a certain level of selectivity. These e-noses are portable, easily scalable, and suitable for detecting VOCs in gas samples. Among other applications, they have been used in the analysis of exhaled breath based on electrochemical detection (Vadera and Dhanekar, 2025).

In the case of plasticizer determination, chromatographic techniques such as gas chromatography (GC) or liquid chromatography (LC) are predominantly employed (Miranda et al., 2025). An alternative approach is capillary electrophoresis (CE), including capillary zone electrophoresis (CZE), which separates and quantifies analytes based on molecular size, charge–to - mass ratio, and isoelectric point (Bernard et al., 2014). Unfortunately, these methods require highly qualified personnel and expensive laboratory equipment and, moreover, are not suitable for field applications.

Taking this into account, it seems important to search for relatively simple, non-instrumental methods that can be used for at least a preliminary assessment of potential threats, especially in situations where access to highly specialized laboratories is limited. A recent example highlighting the necessity of continuous monitoring of environmental samples is the train accident in the Czech Republic (February 2025), which resulted in the release of toxic benzene (a VOC) into the environment and the subsequent contamination of a nearby lake. Such incidents clearly underscore the urgent need to develop alternative detection methods that are portable, easy to operate and suitable for use outside laboratory settings. This presents a particular challenge, and reliable detection requires innovative approaches (BBC News, 2025).

In this summary we intend to present exemplary alternative, sensors approaches potentially useful for determining organic liquids–pollutants - present in water samples. However, we do not aim to provide a comprehensive review of available sensors for these class of analytes. Instead, we aim to highlight the benefits and drawbacks of some of the approaches proposed. Despite the progress achieved in the development of optical sensors for VOC and plasticizer detection, several fundamental limitations still remain. Most existing studies focus on improving sensitivity under controlled laboratory conditions, often neglecting issues of selectivity, photostability, and real-sample applicability. Furthermore, little attention has been given to the long-term performance of sensing materials under variable humidity, temperature, and complex sample compositions typical of real environments. As a result, many reported sensors demonstrate impressive analytical parameters but fail to meet the practical requirements of continuous or on-site monitoring. Our perspective emphasizes that addressing these gaps requires a shift from purely performance-oriented designs toward materials and sensing strategies that ensure robustness, reproducibility, and environmental safety. “Turn-on” fluorescence sensors offer a feasible pathway toward achieving this goal. We believe that by integrating functional materials with rational sensor design, it is possible to fulfill the current gap between laboratory demonstrations and real applications. We predict that among challenges, regarding real-sample application, the following will be the most important: (i) optimization material engineering of the receptor part, (ii) looking for new fluorophores, (iii) improvement of sensitivity (iv) increasing biocompatibility and (v) improving sensor performance in complex matrices, not only in model samples.

## 2 Volatile organic compounds and plasticizers

VOCs are carbon-based substances that exist in solid or liquid form and can transfer into the gas phase through evaporation at a pressure and temperature equal to or higher than 0.01 kPa and 20 °C, respectively (Mangotra and Singh, 2024). VOCs are commonly used in the production of pharmaceuticals, paints, solvents, and refrigerants (Figure 1). Due to industrial growth, the average annual emission of anthropogenic VOCs is expected to increase significantly in the coming years (Zhou et al., 2023). VOCs are classified into major groups: aromatic hydrocarbons, halogenated organic compounds, organic sulfides and sulfinyl compounds, ketones, and esters. Although they generally exhibit low chemical reactivity and limited solubility in water, VOCs can have toxic effects on human health and the environment (Baskaran et al., 2024; Shen et al., 2024; Yang et al., 2025).

Because of their relatively high volatility at room temperature, VOCs often occur as vapors or liquids, which can trigger reactions in the respiratory system and contribute to an increased risk of asthma and other pulmonary diseases. Individuals with prolonged exposure in poorly ventilated or enclosed spaces, such as factory or warehouse workers, are particularly at risk (Irga et al., 2024). The global increase in VOC production contributes to the release of these compounds into the environment, leading to serious pollution issues, including contamination of drinking water and disruption of microbial ecosystems. For instance, the EPA has set New Source Performance Standards (NSPS) and National Emission Standards for Hazardous Air Pollutants (NESHAP) to reduce harmful air pollutants and to limit the release of VOCs from refinery operations (USEPA, 2016). Growing concerns have driven interest in simple, sensor-based systems that enable point-of-need VOC detection. However, the detection and quantification of VOCs remain a major challenge in chemical analysis, particularly in environmental monitoring (Li et al., 2021).

Plasticizers, as low - volatility organic compounds, are also widely used in industrial production, primarily in the manufacturing of plastics (Qadeer et al., 2022). Their emission can have a toxic impact on environmental wellbeing (Qadeer et al., 2024). The most commonly used are phthalate plasticizers. Due to their low volatility and reactivity, they have a high potential for bioaccumulation, and their carcinogenicity has been confirmed. As a result, they have been classified as hazardous substances (Flora et al., 2025; Qadeer et al., 2022; Qadeer et al., 2024). Similarly to many VOCs, most plasticizers exhibit low electrochemical activity and lack of optical activity in the visible light range. Consequently, their detection using simple or non-specialized methods remains challenging (Hasan et al., 2025).

## 3 Turn-on fluorescence sensors

Several fluorescence-based strategies have been proven to be highly efficient in both qualitative and quantitative analysis of VOCs and plasticizers in environmental samples. Since the field of fluorometric sensors for determining plasticizers or VOCs is very broad, we highlight here only turn-on fluorescence-based detection strategies. In such methods, the analytical signal increases with rising analyte concentration. Sensors using turn-on fluorescence emission have many advantages over other optical sensors (Bui et al., 2022). They minimize interference from other ions or molecules in real environmental samples, leading to more accurate measurements, and reduce the risk of false signals. Furthermore, the turn-on mechanism provides an easily visualized signal, making it particularly suitable for quantitative analysis. These sensors also simplify the detection process, often requiring fewer steps compared to other methods.

The detection strategies rely on the interaction of fluorometric dyes, embedded in polymeric matrices (in the form of nanoparticles or nanofibers), with target analytes. Such mechanisms often involve dye/polymer disaggregation under specific conditions and are characterized by high sensitivity within low concentration ranges (ppm level). The proposed approaches show potential for real-time monitoring of hazardous organic pollutants in environmental and biomedical applications, offering cost-efficient, simple, and portable alternatives to conventional laboratory techniques. Most classical fluorophores exhibit strong fluorescence properties when well dispersed in solution, whereas their fluorescence intensity decreases in the solid or aggregated state (Figure 1) (Meher et al., 2022). This phenomenon, known as aggregation-caused quenching (ACQ), has been documented since Förster’s discovery of the concentration quenching effect in 1954 (Förster and Kasper, 1954; Mei et al., 2015). It arises from interactions of the aromatic rings of luminophores due to strong intermolecular π–π stacking. When a fluorophore is dissolved in a good solvent (e.g., one of the VOCs or plasticizers), its dilute solution shows strong luminescence. However, with a gradual increase in the fraction of a poor solvent, fluorescence becomes weaker.

Another research direction is based on an alternative detection mechanism. In 2001, Tang et al. introduced the concept of aggregation-induced emission (AIE), the opposite of the ACQ effect. AIE fluorophores are weakly luminescent in solution but become strongly emissive in their aggregated state (Hong et al., 2009; Mei et al., 2015). The advantages of AIE lie in high emission efficiency in the aggregated state, excellent photostability, and extremely low background noise. Therefore, changes in the degree of aggregation of selected dyes, according to either the ACQ or AIE mechanism, lead to fluorescence changes upon contact with various VOCs or plasticizers, opening up possibilities for tracking their concentration in samples.

**FIGURE 2:**
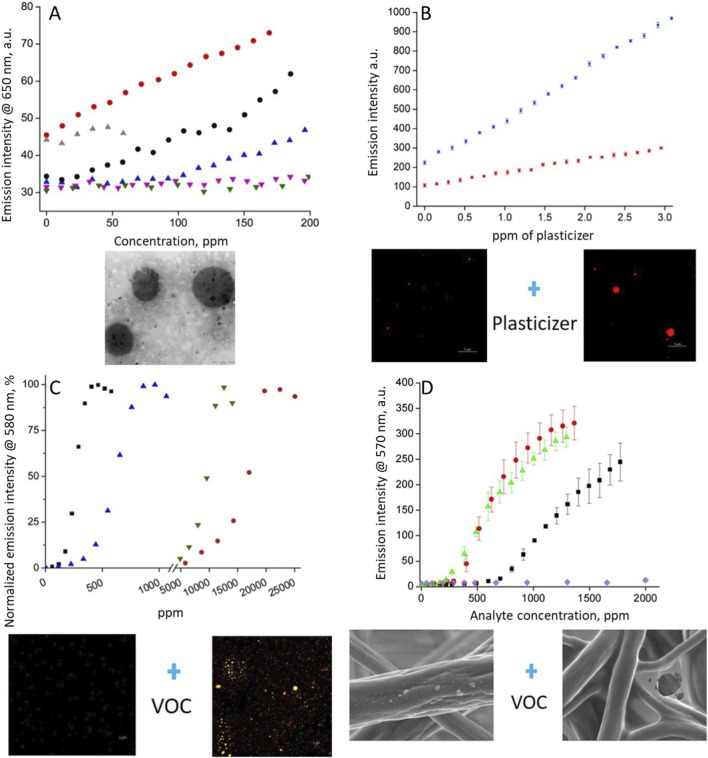
**(A)** Calibration curves recorded in model analytes (●) styrene, (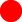) m-xylene, (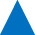) toluene, (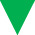) chloroform, (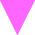) ethanol and (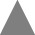) benzene and TEM image of PeHT nanoparticles (reproduced with permission from Kałuża et al., 2022). **(B)** Calibration curves recorded for different concentration of the plasticizer: (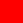) DOS or (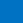) NPOE and confocal microscopy images of PMAO(NR) nanoparticles before and after contact with plasticizer (reproduced with permission from Kisiel et al., 2020a). **(C)** Selectivity of PMAO(POT) nanosponge system for model solvents tested (■) xylene, (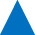) toluene, (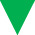) chloroform, (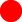) methylene chloride and confocal images of nanosponge sample in the absence and in the presence of xylene. (reproduced with permission from Kisiel et al., 2020b). **(D)** Calibration curves recorded for various analytes: (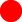) styrene, (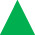) m-xylene, (■) toluene, (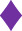) chloroform and SEM images of nanofiber mat before and after contact with VOCs. (reproduced with permission from Kaczmarczyk et al., 2022).

An innovative approach to VOC detection via the ACQ mechanism relies on variations in the fluorescence spectrum of the conducting polymer poly (3-hexylthiophene-2,5-diyl) (PeHT) (Kałuża et al., 2022). PeHT in a good solvent (e.g., THF) shows an intense emission spectrum with a maximum around 570 nm. Upon introducing the THF-dissolved polymer into an aqueous phase considered a poor solvent, PeHT undergoes aggregation, forming polymer nanoparticles. As a result, strong fluorescence quenching is observed, along with the appearance of two new emission maxima around 650 and 720 nm. Upon the addition of styrene (VOC) to the aqueous suspension of PeHT nanoparticles, the compound, due to its high lipophilicity, preferentially localizes within the polymer particles. This leads to the formation of solvent nanodroplets inside the PeHT structure and disaggregation of polymer chains, resulting in an increase in fluorescence intensity. For these nanoparticles, a linear relationship between emission intensity and styrene concentration in the range of 10–200 ppm was recorded (Figure 2A). Importantly, the sensor demonstrated high selectivity toward styrene over other VOCs such as toluene, chloroform, m-xylene, benzene, and ethanol. The high sensitivity of PeHT nanoparticles to styrene in solution also enabled monitoring of its concentration in the gas phase, owing to partitioning between the gas and liquid phases. The proposed sensor allowed detection of styrene in the gas phase within the concentration range of 50–2,100 ppm.

Another approach involved the detection of small amounts of plasticizers in water (Kisiel et al., 2020a). This method relies on the spontaneous diffusion of analytes such as dioctyl sebacate (DOS) or 2-nitrophenyl octyl ether (o-NPOE) from an aqueous solution into fluorescent polymeric nanoparticles. The interaction of the lipophilic plasticizer with nanoparticles made of poly (maleic anhydride-alt-1-octene) (PMAO), combined with Nile red (NR) dye, leads to an increase in the emission intensity of the solvatochromic dye embedded in the nanostructure. This effect results from changes in local polarity and molecular mobility within the nanostructure (Greenspan and Fowler, 1985; Stelmach et al., 2024; Solorzano-García et al., 2025). Transmission electron microscopy (TEM) revealed aggregation/deaggregation of NR on nanoparticle surfaces before and after exposure to plasticizers (Figure 2B). Moreover, results indicate that the type of plasticizer affects both the degree of nanoparticle swelling and the nature of the dye response, due to differences in polarity, solubility, and interaction with the polymer matrix.

A similar detection method was used for m-xylene determination. This approach requires crosslinked polymeric nanoparticles (PMAO) containing aggregates of polythiophene (Kisiel et al., 2020b). Contact of nanostructures with aqueous dispersions of organic solvents results in a significant change in polymeric nanostructure size. PMAO(POT) spheres dispersed in solution under DLS conditions are characterized by a diameter of about 250 nm. The observed increase in diameter is related to the partitioning of xylene (VOC) into the bulk of nanospheres and the spontaneous formation of PMAO(POT) nanoparticles behaving like sponges that absorb VOCs. The selectivity of this nanosponge approach results from the affinity of VOC analytes to polythiophene and the nanoparticle material. An increase in the emission signal was observed for xylene concentrations of 60 or 120 ppm, depending on the amount of dye introduced into the nanostructure (Figure 2C).

A significant improvement in fluorimetric analysis can be achieved by changing the sample format through the application of nanofiber mats produced by electrospinning. A key advantage of nanofiber mats is their high surface-to-volume ratio, which significantly enhances interaction with analytes despite their macroscopic form. The nanofiber mats were prepared from poly (vinyl chloride) (PVC) containing dispersed NR dye (Kaczmarczyk et al., 2022) (Figure 2D). The detection mechanism is based on partial dissolution or deaggregation of the polymer/dye upon contact with organic solvents, which is monitored using optical methods. PVC was selected as the polymer matrix due to its low solubility in many organic solvents, while still allowing swelling or permeation of certain vapors (Lin et al., 2022). Due to the sensor’s highly developed active surface, the amount of NR dye located on or near the fiber surfaces is substantial, enabling effective interaction with analytes without significantly disturbing the nanofiber structure. The proposed sensor for VOCs present in the aqueous phase exhibited a linear response for analyte concentrations ranging from 200 ppm (m-xylene) and 300 ppm (styrene) up to approximately 1,500 ppm.

## 4 Summary and perspectives

This work presents innovative strategies for the detection of VOCs and plasticizers based on fluorescence turn-on signals generated by the interaction of analytes with functional nanostructures. The proposed sensors represent promising alternatives for rapid, on-site detection of harmful compounds, especially in situations where access to a laboratory is limited. Although notable results have been achieved, significant limitations still need to be addressed (Figure 1).

- We predict that in near future, progress in the field of “turn-on” fluorescence sensors for VOC and plasticizer detection will largely depend on advances in molecular design and material engineering. One of the directions of development may be expanding the functionality of nanofibers. Electrospun nanofibers possess various structural, chemical, and mechanical properties that make them suitable for detecting organic pollutants (Agarwal et al., 2025; Behroozi et al., 2023; Oleiwi et al., 2025). Functionalizing their surface is a promising approach to enhancing selectivity, which is often insufficient in currently available sensors. Moreover, mats can be prepared from materials with different wettability, allowing controlled interaction with analytes based on polarity. Porous electrospun nanofibers are one of the most important achievements in nanomaterials research due to their unique structure and function. The use of porous nanostructures can further increase selectivity by enabling interaction only with specific analytes. The another promising direction involves the rational design of functional polymer matrices capable of both capturing analytes and modulating fluorescence in a controllable way.
- Particular attention should be directed toward the development of new fluorophores with high photostability, low toxicity, and tunable emission wavelengths. The use fluorophores and hybrid materials combining organic and inorganic components may offer an effective route to enhance sensitivity and selectivity in complex environmental matrices. Moreover, combining fluorescence-based detection with other signal transduction mechanisms—such as electrochemical—could yield hybrid systems with superior analytical performance.
- In the case of environmental samples, such as those collected after environmental disasters, concentrations may significantly exceed applicable standards, and currently available sensors may be ineffective in detecting pollutants. To achieve tunable sensitivity, sensors can be designed in various nanostructured forms. Depending on whether they are capsules or core - shell spheres, it is possible to fine-tune the sensor’s response according to the needs (Bakker et al., 1997; Woźnica et al., 2014; Woźnica et al., 2017). Tailoring the response of fluorimetric sensors by influencing analyte transport into the probe, or by confining the reaction zone to the sensor surface, can result in a broad linear range of responses, which is crucial in the case of samples with unknown concentrations. On the other hand, in some cases the concentrations of plasticizers or VOCs may be very low, in the ppb range. To overcome this challenge, multifunctional electrospun mats can be used for preconcentration of analytes (Háková et al., 2019; Yahaya et al., 2022). Such preconcentration steps are essential to achieve the required low detection limits for ppb-level analysis.
- At the same time, the application of green, biodegradable, or biocompatible polymers will be essential to ensure environmental safety and long-term sensor stability. Future research should focus on the development of low - cost, miniaturized, and user-friendly sensors, as well as environmentally friendly platforms for real - time monitoring of toxic organic compounds in environmental samples. Another important aspect may be the integration of sensors with artificial intelligence, enabling comparison of results with available databases. Such systems could also provide information about sensor saturation and predict sensor lifetime, contributing to more reliable and long-term applications.
- From the device engineering perspective, future sensors should be lightweight, flexible, and suitable for integration with microfluidic systems. Coupling fluorescence sensing with miniaturized optical components (LEDs, photodiodes, or optical fibers) may facilitate the creation of portable analytical devices capable of real-time monitoring. Finally, a major challenge lies in the integration of data-driven approaches. Advanced algorithms based on machine learning can compensate for environmental interferences, and support predictive maintenance of sensors. The connection of smart materials, sustainable design, and digital analytics may ultimately lead to the development of platforms for the continuous monitoring of hazardous organic pollutants.

## Data Availability

The original contributions presented in the study are included in the article/supplementary material, further inquiries can be directed to the corresponding author.
